# Ubiquitin-specific protease 4 controls metastatic potential through β-catenin stabilization in brain metastatic lung adenocarcinoma

**DOI:** 10.1038/srep21596

**Published:** 2016-02-17

**Authors:** Su Jin Hwang, Hye Won Lee, Hye Ree Kim, Hong Lee, Chang Hoon Shin, Sun-Il Yun, Dong Heon Lee, Duk-Hwan Kim, Kyeong Kyu Kim, Kyeung Min Joo, Hyeon Ho Kim

**Affiliations:** 1 Department of Health Sciences and Technology, Samsung Advanced Institute for Health Sciences and Technology, Sungkyunkwan University, Seoul, Korea; 2 Department of Urology, Samsung Medical Center, Sungkyunkwan University School of Medicine, Seoul, Korea; 3 Department of Neurosurgery, Institute for Refractory Cancer Research, Samsung Medical Center, Sungkyunkwan University School of Medicine, Seoul, Korea; 4 Department of Anatomy and Cell Biology, Sungkyunkwan University School of Medicine, Suwon, Korea; 5 Center for Genome Research, Samsung Medical Center, Sungkyunkwan University School of Medicine, Seoul, Korea; 6 Department of Molecular Cell Biology, Sungkyunkwan University School of Medicine, Suwon, Korea; 7 Samsung Biomedical Research Institute, Samsung Medical Center, Seoul, Korea

## Abstract

Brain metastasis is the most common type of intracranial cancer and is the main cause of cancer-associated mortality. Brain metastasis mainly originates from lung cancer. Using a previously established *in vitro* brain metastatic model, we found that brain metastatic PC14PE6/LvBr4 cells exhibited higher expression of β-catenin and increased migratory activity than parental PC14PE6 cells. Knockdown of β-catenin dramatically suppressed the motility and invasiveness of PC14PE6/LvBr4 cells, indicating β-catenin is involved in controlling metastatic potential. Since β-catenin protein was increased without a significant change in its mRNA levels, the mechanism underlying increased β-catenin stability was investigated. We found that ubiquitin-specific protease 4 (USP4), recently identified as a β-catenin-specific deubiquitinylating enzyme, was highly expressed in PC14PE6/LvBr4 cells and involved in the increased stability of β-catenin protein. Similar to β-catenin knockdown, USP4-silenced PC14PE6/LvBr4 cells showed decreased migratory and invasive abilities. Moreover, knockdown of both USP4 and β-catenin inhibited clonogenicity and induced mesenchymal-epithelial transition by downregulating ZEB1 in PC14PE6/LvBr4 cells. Using bioluminescence imaging, we found that knockdown of USP4 suppressed brain metastasis *in vivo* and significantly increased overall survival and brain metastasis-free survival. Taken together, our results indicate that USP4 is a promising therapeutic target for brain metastasis in patients with lung adenocarcinoma.

Brain metastasis is a main cause of cancer-related morbidity and mortality and occurs in approximately 20–40% of patients with advanced cancers. Lung cancer is one of the most malignant human cancers and is divided into 2 main types: small cell lung cancer (SCLC) and non-small cell lung cancer (NSCLC). SCLC is known to respond better to chemotherapy and radiotherapy; however, NSCLC, which accounts for 80–85% of all lung cancers, is very difficult to treat despite great advances in the development of therapeutics for lung cancer^1^.

The canonical Wnt/β-catenin pathway is highly conserved and frequently dysregulated in many cancers. Growing evidence has demonstrated that the Wnt/β-catenin pathway plays a critical role in the development of NSCLC. Several components of the Wnt/β-catenin pathway and β-catenin target genes including c-Myc, cyclin D1, VEGF-A, MMP-7, and survivin are overexpressed in NSCLC^2^. Moreover, nuclear β-catenin is associated with epidermal growth receptor (EGFR) mutations^3^ and resistance to gefitinib^4^. Aberrant activation of β-catenin signaling is also known to participate in the epithelial-mesenchymal transition (EMT), which is a key step in metastatic processes and plays an important role in the dissemination of cancer cells^5^. Although mutations in β-catenin or its regulator, adenomatous polyposis coli are not frequently found in lung cancer, several studies have demonstrated that Wnt/β-catenin signaling is closely associated with tumorigenesis, prognosis, and resistance therapy^2^.

Cytoplasmic β-catenin is maintained at low levels through ubiquitin-mediated degradation. Ubiquitination/proteasome degradation of β-catenin is initiated by phosphorylation of S45 by casein kinase 1α (CK1α) and subsequently by constitutively active glycogen synthase kinase 3 (GSK3) at S33, S37, and T41. Phosphorylated β-catenin is recognized by E3 ligase, ubiquitinylated, and degraded by the proteasome. In addition to ubiquitination, a deubiquitinating mechanism also plays an essential role in the regulation of β-catenin. Deubiquitinating enzymes (DUBs) remove covalently bound ubiquitin from target proteins and thereby regulate their activity and abundance^6^. Several DUBs have been reported to be associated with the Wnt/β-catenin signaling pathway. USP8/UBPY is reported to activate the Wnt/β-catenin pathway by targeting Frizzled G-protein coupled protein^7^. In contrast, USP34 functions as a negative regulator by triggering the degradation of Axin^8^.

Through left ventricle (LV) injection of PC14PE6 lung adenocarcinoma cells, we previously isolated brain metastatic cells known as PC14PE6/LvBr4 cells^9^. The brain metastatic PC14PE6/LvBr4 cells exhibited higher invasiveness than their parental PC14PE6 cells. In this study, we investigated the molecular mechanism by which PC14PE6/LvBr4 cells exhibit higher metastatic potential than their parental cells. Based on proteomic analysis, we found that β-catenin is highly expressed in PC14PE6/LvBr4 cells, and USP4, which was recently identified as a β-catenin-specific DUB^10^, is responsible for increased expression of β-catenin. Knockdown of β-catenin and USP4 suppressed the metastatic potential, including migration and invasion and inhibited the *in vivo* brain metastasis of PC14PE6/LvBr4 cells.

## Results

### Brain metastatic PC14PE6/LvBr4 cells exhibited higher expression of β-catenin and increased migratory activity

**Figure 1:**
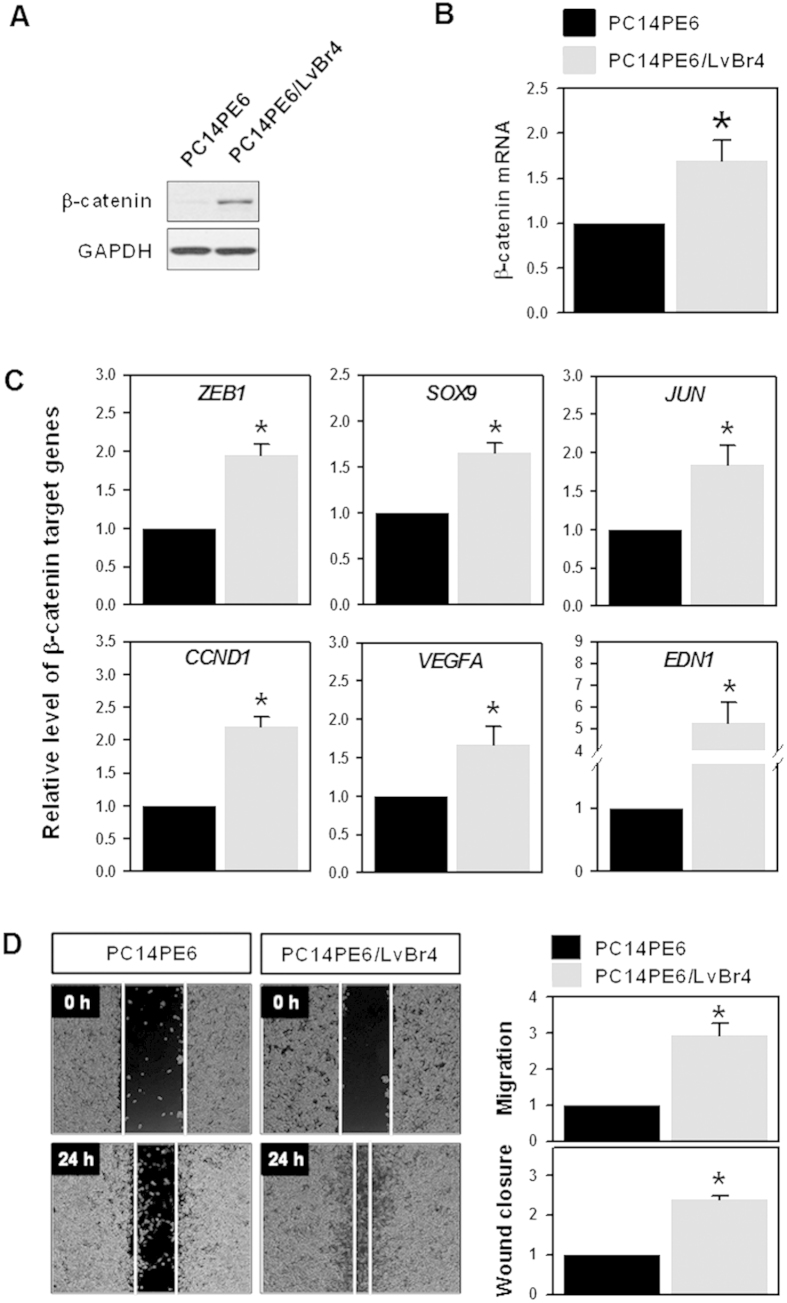
Brain metastatic PC14PE6/LvBr4 cells exhibited higher expression of β-catenin and metastatic potential than parental PC14PE6 cells. (**A,B**) The expression level of β-catenin protein and mRNA was determined by western blotting (**A**) and RT-qPCR (**B**), respectively. (**C**) The expression level of β-catenin target genes was assessed by RT-qPCR. (**D**) To compare the migratory activity between PC14PE6 and PC14PE6/LvBr4 cells, an *in vitro* wound closure assay and Transwell migration assay were performed as described in the Materials and Methods. Data are means and standard deviation from more than three independent experiments. *p < 0.05.

We established an *in vitro* brain metastasis model through left ventricle (LV) injection of lung adenocarcinoma PC14PE6 cells, and isolated brain metastatic PC14PE6/LvBr4 cells^9^. Compared with parental PC14PE6 cells, we found that PC14PE6/LvBr4 cells exhibited higher migratory and invasive activities compared to parental PC14PE6 cells. To investigate the molecular mechanism underlying this higher metastatic potential of PC14PE6/LvBr4 cells, we compared the level of various signaling molecules using phospho-kinases proteome profiler (see Supplementary material online, Fig. S1). We found that β-catenin protein levels were higher in PC14PE6/LvBr4 cells than that in parental PC14PE6 cells (Fig. 1A); however, there was no significant change in β-catenin mRNA (Fig. 1B). To verify the increased expression of β-catenin, the expression level of β-catenin target genes was examined by reverse transcription-quantitative polymerase chain reaction (RT-qPCR). All tested β-catenin target genes, including zinc finger E-box-binding homeobox 1 (*ZEB1*), sex determining region Y-box 9 (*SOX9*), c-Jun (*JUN*), cyclin D1 (*CCND1*), vascular endothelial growth factor A (*VEGFA*), and endothelin 1 (*EDN1*), were highly expressed in PC14PE6/LvBr4 cells. It was previously reported that brain metastatic PC14PE6/LvBr4 cells show higher invasiveness than parental PC14PE6 cells^9^. In addition to invasiveness, PC14PE6/LvBr4 cells showed increased migration ability compared to PC14PE6 cells (Fig. 1D), indicating that the established brain metastatic cells, PC14PE6/LvBr4, acquire higher metastatic potential including migration and invasion upon dissemination into the brain. To compare the level of destruction complex components between parental PC14PE6 and brain metastatic PC14PE6/LvBr4 cells, we checked the expression level of APC and GSK3β. As shown in Supplementary figure S2, their expression level was almost same, indicating that destruction complex for β-catenin is not involved in the increased expression of β-catenin. Considering the increased level of total b-catenin, we also found that phosphorylation of β-catenin is not responsible for the increase of β-catenin.

### Knockdown of β-catenin suppressed the metastatic potential of brain metastatic PC14PE6/LvBr4 cells

**Figure 2:**
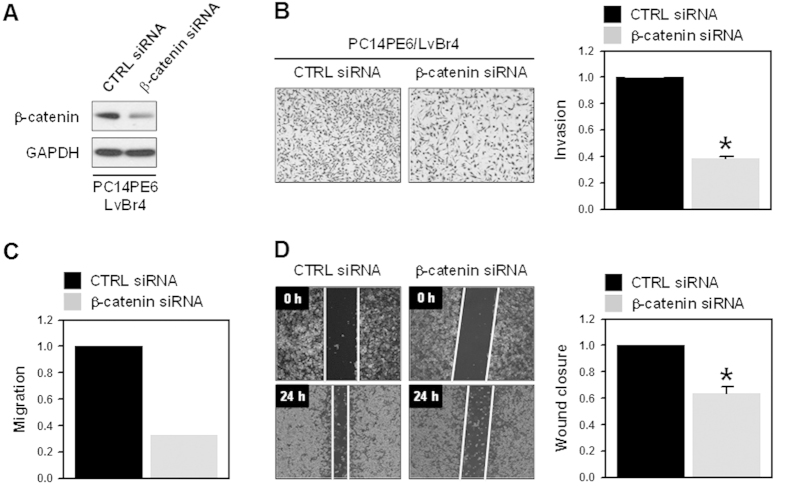
Knockdown of β-catenin suppressed metastatic potential of brain metastatic PC14PE6/LvBr4 cells. (**A**) To evaluate the effect of β-catenin silencing on metastatic potential, brain metastatic PC14PE6/LvBr4 cells were transfected with control (CTRL) or β-catenin-specific siRNA for 48 h. The expression level of β-catenin was determined by western blotting. (**B–D**) Metastatic potential including invasion (**B**) and migration (C: Transwell, D: wound closure) was determined as described in the Materials and Methods. Data are means and standard deviation from more than three independent experiments. *p < 0.05.

To investigate the role of β-catenin in the regulation of metastatic potential, PC14PE6/LvBr4 cells were transfected with control (CTRL) or β-catenin-specific siRNA. The expression level of β-catenin was determined by western blot analysis (Fig. 2A). Transient transfection of β-catenin-targeting siRNA significantly reduced the invasiveness (Fig. 2B) and migration (Transwell migration assay and wound closure assay in Fig. 2C,D, respectively), suggesting that increased β-catenin expression is closely associated with a higher metastatic potential of PC14PE6/LvBr4 cells. The effect of β-catenin on the invasiveness was verified by performing the invasion assay using parental PC14PE6 and H1299 cells (see Supplementary material online, Fig. S3 and Fig. S4, respectively).

### USP4 increased the level of β-catenin by inducing its deubiquitinylation

**Figure 3:**
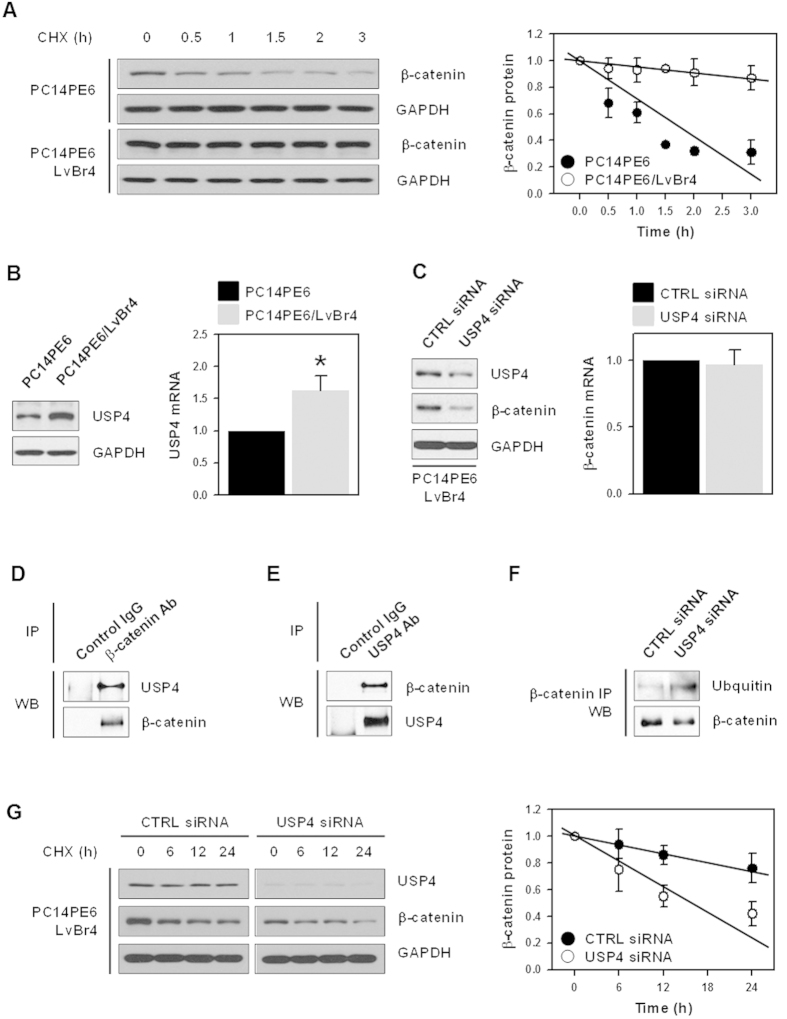
USP4 regulated the expression of β-catenin by controlling its protein stability. (**A**) To compare the β-catenin protein stability, PC14PE6 and PC14PE6/LvBr4 cells were treated with cycloheximide (20 μg/mL) and harvested at the indicated times. Whole cell lysates were prepared, and the level of β-catenin protein was determined by western blotting. The stability of β-catenin was assessed by image analysis. (**B**) The expression level of USP4 in PC14PE6 and PC14PE6/LvBr4 cells was examined by western blotting (left panel) and RT-qPCR (right panel). (**C**) To determine whether USP regulates the expression of β-catenin, brain metastatic PC14PE6/LvBr4 cells were transfected with control (CTRL) or USP4-specific siRNA for 48 h. The whole cell extract was prepared and the expression level of USP4 and β-catenin was determined by western blotting. (**D,E**) To evaluate the direct interaction between USP4 and β-catenin, whole cell lysates were prepared using RIPA buffer, and equal amounts of protein were incubated with appropriate control IgG or an antibody against USP4 (**D**) or β-catenin (**E**). The level of β-catenin and USP4 in IP materials was assessed by western blotting. (**F**) To check the effect of USP4 silencing on the ubiquitination of β-catenin, PC14PE6 cells were transfected with control (CTRL) or USP4-specific siRNA for 48 h. Whole cell lysates were prepared using RIPA buffer and incubated with appropriate control IgG and β-catenin antibody. The levels of β-catenin and ubiquitin were assessed by western blotting. (**G**) To compare the protein stability of β-catenin, parental PC14PE6 and brain metastatic PC14PE6/LvBr4 cells were treated with cycloheximide (40 μg/mL) and harvested at the indicated times. The stability of β-catenin was determined as described above. Data are means and standard deviation from more than three independent experiments. *p < 0.05.

We found that β-catenin is highly expressed in PC14PE6/LvBr4 cells, but its mRNA levels were not significantly changed (Fig. 1A,B); thus, we compared the stability of β-catenin. PC14PE6 and PC14PE6/LvBr4 cells were treated with cycloheximide and then harvested at the indicated times. β-Catenin protein levels were assessed by western blotting and were quantified by image analysis using the Image J program. While the level of β-catenin protein was dramatically decreased after 1.5 h post-treatment in PC14PE6 cells, that in PC14PE6/LvBr4 cells was sustained until 3 h (Fig. 3A). These results suggest that the higher level of β-catenin in PC14PE6/LvBr4 cells resulted from increased protein stability. Since our colleagues recently identified USP4 as a β-catenin-specific DUB^10^, we compared the levels of USP4 between PC14PE6 and PC14PE6/LvBr4 cells (Fig. 3B). Similar to β-catenin, USP4 was highly expressed in PC14PE6/LvBr4 cells. To examine whether USP4 regulates the expression of β-catenin, PC14PE6/LvBr4 cells were transfected with CTRL or USP4-specific siRNA for 48 h. Transient transfection of USP4 siRNA effectively reduced the expression of USP4 protein (Fig. 3C). Interestingly, it was also found that knockdown of USP4 decreased the level of β-catenin protein without changes in its mRNA level (Fig. 3C). Thus, we examined the direct interaction between USP4 and β-catenin in our metastatic model system. Whole cell lysates (WCLs) obtained from PC14PE6/LvBr4 cells were incubated with an appropriate control IgG or an antibody against USP4 or β-catenin. The levels of USP4 and β-catenin in immunoprecipitated (IP) materials were assessed by western blotting. USP4 was detected in β-catenin IP materials (Fig. 3D), and β-catenin was found in USP4 IP materials (Fig. 3E), suggesting that USP4 and β-catenin directly bind to each other. To examine the effect of USP4 silencing on the ubiquitination of β-catenin, PC14PE6/LvBr4 cells were transfected with CTRL or USP4-specific siRNA for 48 h and WCLs were prepared for IP experiments. After incubation of WCLs with the appropriate control IgG or β-catenin antibody, ubiquitinylated β-catenin was detected using an ubiquitin-specific antibody (Fig. 3F). We found that knockdown of USP4 decreased the ubiquitination of β-catenin, indicating that USP4 is involved in regulating β-catenin expression through the ubiquitination of β-catenin. To investigate the effect of USP4 on β-catenin protein stability, PC14PE6/LvBr4 cells were transfected with CTRL and USP4 siRNA and then resuspended into 35-mm dishes. After treatment with 40 μg/ml cycloheximide, the cells were harvested at the indicated times. The level of β-catenin was determined by western blotting and quantified by image analysis. β-Catenin protein stability was significantly lower in USP4-silenced PC14PE6/LvBr4 cells than in PC14PE6 cells (Fig. 3G), demonstrating that USP4 positively regulated β-catenin expression in metastatic lung cancer cells.

### Knockdown of USP4 suppressed metastatic potential of brain metastatic PC14PE6/LvBr4 cells

**Figure 4:**
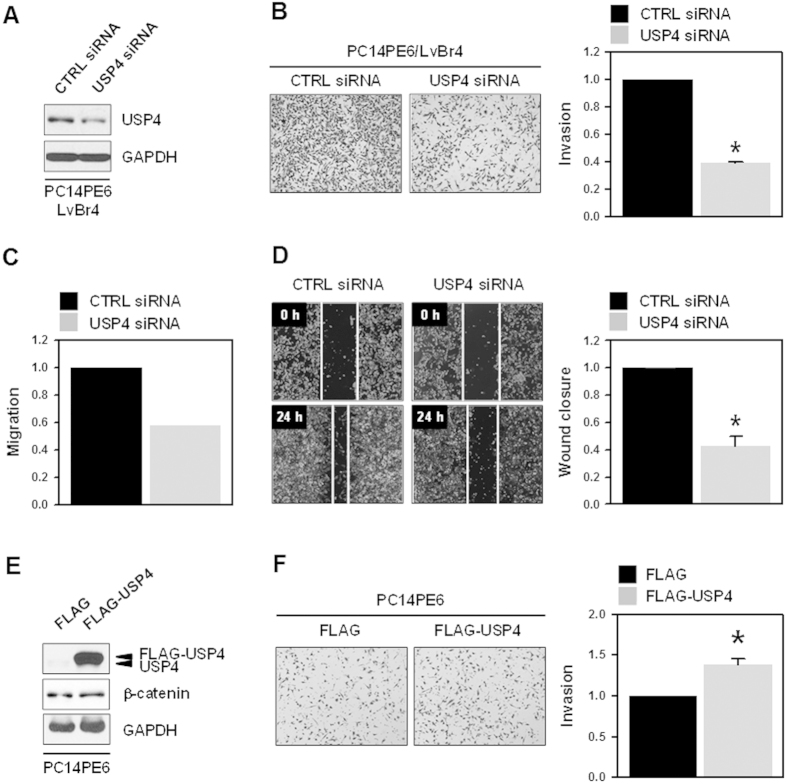
Knockdown of USP4 inhibited metastatic potential of brain metastatic PC14PE6/LvBr4 cells. (**A**) To assess the function of USP4 in metastatic potential, PC14PE6/LvBr4 cells were transfected with control (CTRL) or USP4-specific siRNA for 48 h. The expression level of USP4 was determined by western blotting. (**B–D**) Metastatic potential including invasion (**B**) and migration (**C**: Transwell, **D**: wound closure) was determined as described in the Materials and Methods. (**E,F**) To check whether USP4 overexpression upregulates β-catenin expression and increases invasiveness, PC14PE6 cells were transfected with blank (FLAG) or USP4-overexpressing vector (FLAG-USP4). The level of USP4 and β-catenin was determined by Western blot (**E**) and invasiveness was assessed using a Matrigel Invasion Chamber (**F**). Data are means and standard deviation from more than three independent experiments. *p < 0.05.

Based on the above results, we found that β-catenin was involved in the metastatic potential of PC14PE6/LvBr4 cells and that USP4 upregulated the expression of β-catenin by deubiquitinylating β-catenin. Therefore, we evaluated the effect of USP4 silencing on the invasive and migratory activities. PC14PE6/LvBr4 cells were transfected with CTRL or USP4-specific siRNA. USP4 was effectively downregulated by transient transfection of specific siRNA (Fig. 4A). Knockdown of USP4 significantly inhibited the invasive activity of PC14PE6/LvBr4 cells (Fig. 4B). In addition, migratory activity was also inhibited by silencing of USP4 (Transwell migration assay and wound closure assay in Fig. 4C,D, respectively). Based on these results, we found that USP4 regulated the migratory and invasive abilities of PC14PE6/LvBr4 cells, demonstrating that USP4/β-catenin controls the metastatic potential of lung adenocarcinoma cells. To examine whether USP4 overexpression increases β-catenin expression and invasiveness, PC14PE6 cells were transfected with blank (FLAG) and USP4-overexpression vector (FLAG-USP4). USP4-overexpressed cells showed slight increase of β-catenin expression (Fig. 4E) and thus more invasive ability (Fig. 4F). In addition to transient transfection of USP4 siRNA, stable knockdown of USP4 with specific shRNA also downregulated b-catenin and suppressed invasive and migratory abilities of PC14PE6/LvBr4 cells (see Supplementary material online, Fig. S6). These results indicate that USP4 is required for increased expression of β-catenin and gain of more metastatic properties including invasion and migration.

### XAV939 decreased β-catenin expression and inhibited metastatic potential in brain metastatic PC14PE6/LvBr4 cells

**Figure 5:**
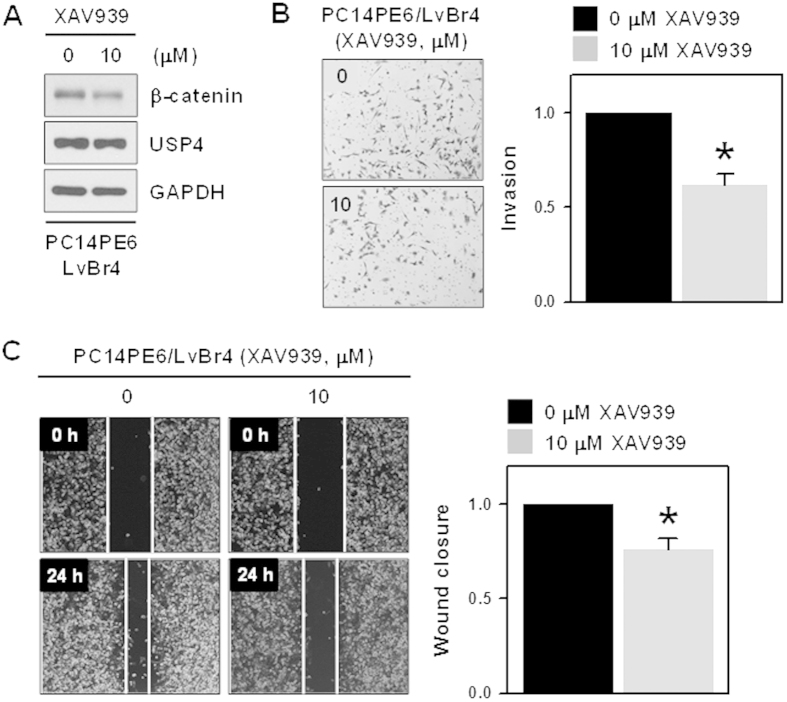
XAV939 decreased β-catenin expression and inhibited the invasive and migratory abilities in brain metastatic PC14PE6/LvBr4 cells. (**A**) PC14PE6 cells were treated with 10 μM XAV939 for 24 h and then whole cell lysates were prepared with RIPA buffer. The expression level of β-catenin and USP4 was determined by Western blot. (**B–C**) Metastatic potential including invasion (**B**) and migration (C: wound closure) was determined as described in the Materials and Methods. Data are means and standard deviation from more than three independent experiments. *p < 0.05.

XAV939 is identified as a tankyrase inhibitor and reported to selectively inhibit β-catenin-mediated transcription by stabilizing axin, a component of destruction complex for β-catenin. To verify that β-catenin is essential for acquisition of metastatic potential, we tested the effect of XAV939 on β-catenin expression and metastatic potential. PC14PE6/LvBr4 cells treated with XAV939 showed decreased expression of β-catenin; however, USP4 expression was not affected (Fig. 5A). As expected, treatment of XAV939 to PC14PE6/LvBr4 cells suppressed invasive and migratory abilities (Fig. 5B,C, respectively), suggesting that β-catenin is essential for metastatic potential of PC14PE6/LvBr4 cells.

### USP4/β-catenin axis regulated the clonogenicity and epithelial-mesenchymal transition in brain metastatic PC14PE6/LvBr4 cells

**Figure 6:**
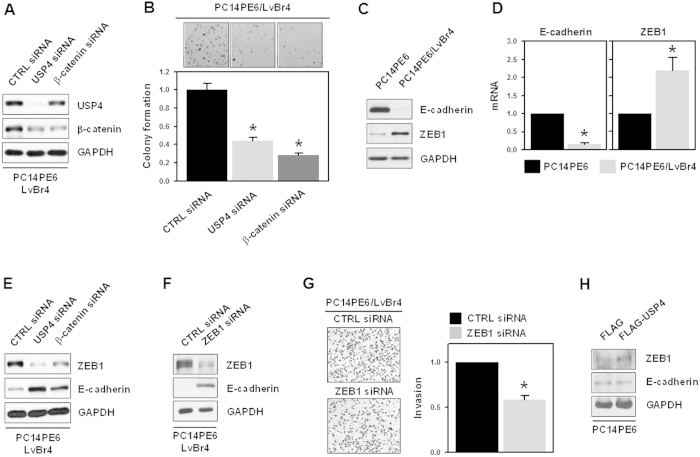
USP4/β-catenin axis regulated the clonogenicity and epithelial-mesenchymal transition in brain metastatic PC14PE6/LvBr4 cells. (**A**) For the colony forming assay, PC14PE6/LvBr4 cells were transfected with control (CTRL) or siRNAs targeting USP4 or β-catenin for 48 h. The level of USP4 and β-catenin was determined by western blotting. (**B**) Transfected cells were seeded into 6-well plates and cultured with complete medium for 2 weeks. After cells were stained with 0.2% crystal violet, the number of stained colonies was counted. (**C,D**) To compare the EMT characteristics between PC14PE6 and PC14PE6/LvBr4 cells, the expression level of E-cadherin and ZEB1 was determined by western blotting (**C**) and RT-qPCR (**D**). (**E**) To examine the effect of USP4 and β-catenin on ZEB1 expression, PC14PE6/LvBr4 cells were transfected with control (CTRL) siRNA or siRNAs targeting USP4 or β-catenin for 48 h. The levels of USP4, β-catenin, and ZEB1 were determined by western blotting. (**F,G**) To investigate the effect of ZEB1 silencing on EMT and invasiveness, PC14PE6/LvBr4 cells were transfected with control (CTRL) or ZEB1-specific siRNA for 48 h. The levels of ZEB1 and E-cadherin were determined by western blotting (**F**) and the invasiveness of transfected cells was assessed using a Matrigel Invasion Chamber (**G**). Data are means and standard deviation from more than three independent experiments. *p < 0.05.

To investigate the role of USP4/β-catenin in clonogenicity, PC14PE6/LvBr4 cells were transfected with CTRL or siRNAs targeting USP4 or β-catenin. Transfected cells were seeded into 6-well plates and cultured for 2 weeks. After cells were stained with 0.2% crystal violet, the number of stained colonies was counted. Both siRNAs efficiently downregulated the expression of USP4 and β-catenin (Fig. 6A) and significantly inhibited the clonogenicity of PC14PE6/LvBr4 cells (Fig. 6B).

The EMT is known to be a leading step of the metastatic processes. EMT is characterized by the decreased expression of epithelial markers in contrast to increased expression of mesenchymal markers. Since PC14PE6/LvBr4 cells exhibited higher metastatic activities, we examined the mesenchymal characteristics of PC14PE6/LvBr4 cells by comparing E-cadherin levels. We found that E-cadherin expression was decreased in PC14PE6/LvBr4 cells, which resulted from the increased expression of ZEB1, a transcriptional suppressor of E-cadherin (Fig. 6C,D). Since ZEB1 is known to be a target gene of β-catenin, we evaluated whether the USP4/β-catenin axis regulates EMT processes in PC14PE6/LvBr4 cells. PC14PE6/LvBr4 cells were transfected with CTRL, USP4, or β-catenin siRNA for 48 h and the levels of ZEB1 and E-cadherin were determined by western blotting (Fig. 6E and see also Supplementary material online, Fig. S5). We found that knockdown of both USP4 and β-catenin downregulated the expression of ZEB1 and thereby increased the level of E-cadherin, indicating that the USP4/β-catenin axis is required for the EMT process and involves the regulation of ZEB1. To examine whether ZEB1 regulation by the USP4/β-catenin axis is involved in metastatic potential, PC14PE6/LvBr4 cells were transfected with CTRL or ZEB1-specific siRNA for 48 h. The levels of ZEB1 and E-cadherin were determined by western blotting (Fig. 6F) and invasive activity was assessed using the Matrigel invasion assay (Fig. 6G). We found that ZEB1-specific siRNA effectively decreased the expression of ZEB1 and that knockdown of ZEB1 suppressed the invasiveness of PC14PE6/LvBr4 cells. Our results suggest that USP4/β-catenin axis controls EMT process by induction of the expression of ZEB1 and subsequent downregulation of E-cadherin, which is responsible for acquisition of invasive ability. To verify that USP4 induces EMT process by upregulating β-catenin expression, we checked the expression level of ZEB1 and E-cadherin in USP4-overexpressing PC14PE6 cells (Fig. 6H). USP4 overexpression increased ZEB1 expression and accordingly, the expression level of E-cadherin was decreased. In accordance with increased invasiveness, these results indicate USP4 is involved in EMT process.

### Implications of USP4/β-catenin axis in brain metastasis of lung adenocarcinoma *in vivo*

**Figure 7:**
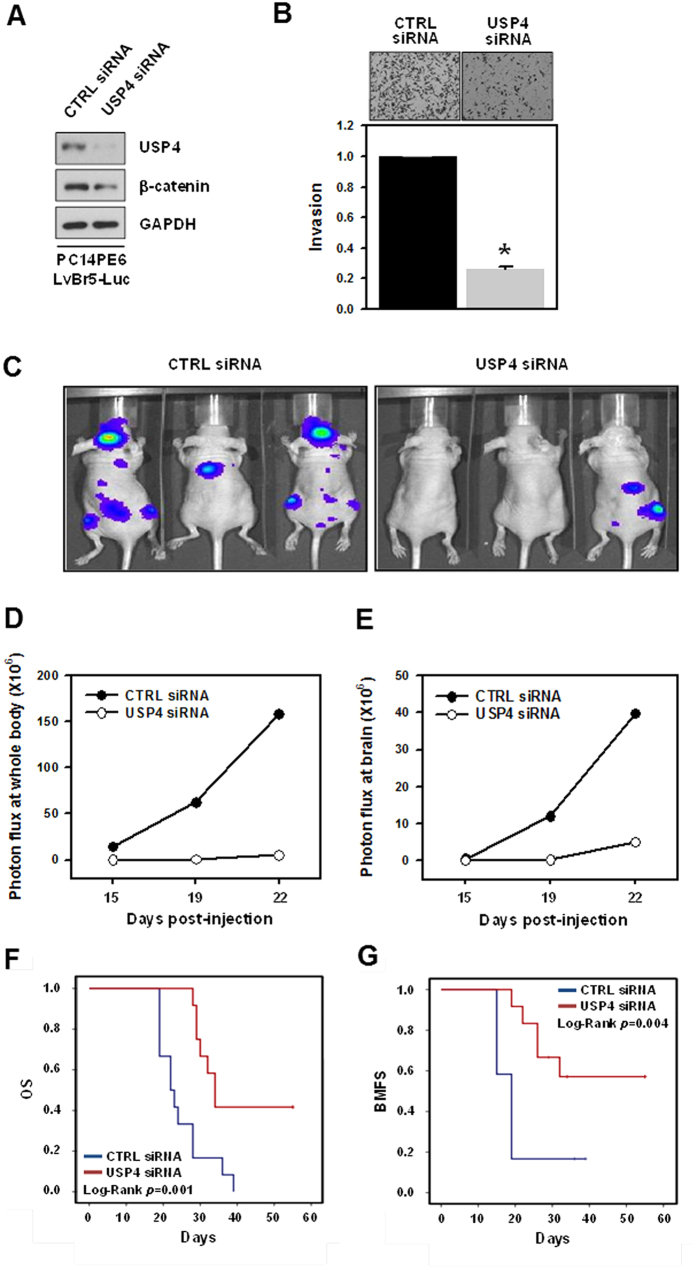
Knockdown of USP4 inhibited brain metastasis and promoted overall survival (OS) and brain metastasis-free survival (BMFS). (**A**) PC14PE6/LvBr5-Luc cells were transfected with control (CTRL) siRNA or USP4-specific siRNA for 48 h. The levels of USP4, β-catenin, E-cadherin, and ZEB1 were determined by western blotting. (**B**) For the invasion assay, equal numbers of transfected cells were inoculated into a Transwell and invaded cells were stained and counted under a microscope. Data are means and standard deviation from more than three independent experiments. (**C**) To investigate the effect of USP4 silencing on brain metastasis *in vivo*, transfected PC14PE6/LvBr5-Luc cells were directly injected into the left ventricle (LV) of the heart. Bioluminescence images were acquired at 22 days post-injection. (**D**,**E**) Incidence of whole body (**D**) and brain metastasis (**E**) were quantified by the luminescent signal at a given time point. (**F,G**) Kaplan–Meier curves and *p*-values for overall survival (OS) and brain metastasis-free survival (BMFS) were analyzed in systemic metastasis models. ^*^p < 0.05.

Our results strongly suggest that USP4 plays a critical role in the control of clonogenicity, migration, and invasion. Therefore, we further investigated the function of USP4 in metastasis *in vivo*. For bioluminescence imaging, PC14PE6/LvBr5-Luc cells were previously established^9^. Prior to the *in vivo* study, we examined the effect of USP4 silencing on the expression of β-catenin and ZEB1. As expected, knockdown of USP4 significantly downregulated the expression of β-catenin (Fig. 7A) and suppressed the invasiveness of PC14PE6/LvBr5-Luc cells (Fig. 7B). Next, we investigated the effect of USP4 silencing on the metastasis of PC14PE6/LvBr5-Luc cells by intracardiac injection (2 × 10^5^ PC14PE6-LvBr5-Luc/CTRL siRNA, N = 10 or PC14PE6-LvBr5-Luc/USP4 siRNA, N = 9). Bioluminescence images were acquired at 22 days post-injection (Fig. 7C) and quantified using the luminescent signals at the indicated time points. Knockdown of USP4 dramatically suppressed the metastasis of PC14PE6/LvBr5-Luc cells to the whole body and brain, as reflected by bioluminescence signals at 19 and 22 days post-injection (Fig. 7D,E). We also found that knockdown of USP4 prolonged overall survival (OS) and brain metastasis-free survival (BMFS) of lung adenocarcinoma-bearing mice (Fig. 7F,G, respectively, all Log rank *p* < 0.001), suggesting that USP4 promotes the metastatic abilities of lung adenocarcinoma cells.

## Discussion

Brain metastasis is associated with the poor prognosis of cancer patients with systemic malignancy and mainly originates from primary lung cancer (40–50%), breast cancer (15–25%), or melanoma (5–20%)^11^,^12^. Brain metastasis represents the most common type of intracranial cancer, occurring ten times more often than primary brain cancer^13^. By analyzing the differentially expressed genes in animal models and human tissue cohorts, several genes, including *CDH2*, *KIFC1*, and *FALZ*, were identified to be highly associated with lung cancer brain metastasis^14^. It was recently reported that DCN-like protein 1, encoded by the *DCUN1D1* gene, is amplified in several types of cancer as an oncogene and was identified as a molecular marker of lung cancer patients at high risk for brain metastasis^15^. Studies examining the molecular signature of brain-specific metastasis have identified α-2, 6-sialyltransferase *ST6GALNAC5* (also known as α-*N*-acetylgalactosaminide), which is overexpressed in brain-trophic, but not in lung- or bone-trophic, metastasized cancer cells^16^. Breast cancer cells showing high expression of ST6GALNAC5 preferentially metastasize into the brain, and knockdown of ST6GALNAC5 diminished the migration of cancer cells across the blood-brain barrier and suppressed brain metastasis in an animal model. Despite widespread efforts to determine the molecular pathways underlying lung cancer-derived brain metastasis, the detailed mechanism remains largely unknown.

Approximately 85% of lung cancer is NSCLC in which the mutation of β-catenin and APS is unusual: however, Wnt/β-catenin signaling is known to be closely associated with lung cancer progression^2^. In lung cancer, the Wnt pathway has been shown to be correlated with brain metastasis. Activation of Wnt signaling occurs through the binding of ligands to its receptors, stabilizing β-catenin, which induces dimerization with the lymphoid enhancer-binding factor (LEF) transcription factor (TCF) family. TCF target genes are closely associated with the prognosis of lung cancer metastasis, and dominant-negative TCFs suppress the brain metastasis of lung cancer cells by regulating LEF1 and the homeobox protein HOXB9^17^. β-Catenin is highly expressed in 51% of lung adenocarcinoma^18^, and its nuclear accumulation and overexpression are reported to be associated with mutations in EGFR^3^ and resistance to gefitinib^4^, respectively. Moreover, activation of Wnt/β-catenin signaling is involved in the stemness of lung cancer and the intrinsic or acquired resistance against various chemotherapeutics^19^. Activation of Wnt/β-catenin is also clinically associated with poor prognosis of patients with advanced lung cancer^20^,^21^,^22^.

Emerging evidence has demonstrated that the ubiquitin/proteasome machinery is a master regulator of the Wnt/β-catenin pathway. Cellular levels of β-catenin are also tightly regulated by phosphorylation-dependent ubiquitination and subsequent proteasomal degradation. 2 main kinases are involved in the phosphorylation of β-catenin: CK1 and GSK3. Prior to GSK3-mediated phosphorylation, a prime kinase CK1 induces the phosphorylation of serine-threonine rich region, and constitutively active GSK3 is able to phosphorylate β-catenin at 2 serine residues. Several β-catenin ubiquitin ligases, including β-transducin repeats-containing proteins^23^, casitas B-lineage lymphoma^24^, and seven in absentia homolog 1^25^, have been identified. In addition to ubiquitination, deubiquitination also plays a critical role in maintaining β-catenin levels. DUBs are capable of removing attached ubiquitin from target proteins and are responsible for recycling the monoubiquitin from unanchored polyubiquitin^26^. Approximately 95 putative DUBs are encoded in the human genome and are divided into five subclasses: ubiquitin-specific proteases (USP), ubiquitin C-terminal hydrolases, Machado-Joseph domain proteases, ovarian tumor related proteases, and JAB1/MPN/Mov34 metalloenzyme^27^. USP proteins are the most characterized DUBs and belong to cysteine protease family. Recently, USP4 was identified as a β-catenin-specific DUB that positively regulates β-catenin signaling in colon cancer^10^. USP4 is reported to participate in diverse signaling pathways by deubiquitinating signaling molecules^28^,^29^. USP4 was found to be involved in the deubiquitination of Ro52^30^, pyruvate dehydrogenase kinase isozyme 1^31^, ARF-binding protein 1^32^, transforming growth factor-β-activated kinase 1^33^, and tumor necrosis factor receptor-associated factor^34^.

The initial key step of the metastatic process is the EMT. During EMT, epithelial cells acquire mesenchymal characteristics, which confer metastatic potential including motility and invasiveness on cancer cells. The expression of E-cadherin is transcriptionally repressed by several transcriptional suppressors such as ZEB1/2, SNAI1/2, and Twist1/2^35^. As an inducer of EMT, ZEB1 not only represses the expression of epithelial markers, but also induces an increase in mesenchymal markers. ZEB1 is also associated with tumorigenesis, stemness, and resistance^36^,^37^,^38^. Several studies have demonstrated that the aberrant activation of β-catenin signaling participates in the EMT process^39^. Activation of the canonical Wnt pathway phosphorylates β-catenin and thus induces the translocation of β-catenin into the nucleus, where it activates the transcription of EMT-related genes. Among the target genes involved in the ENT process, ZEB1 is reportedly a direct target gene of β-catenin. β-Catenin/TCF4 activates the transcription of ZEB1 by directly binding to the ZEB1 promoter region^40^. ZEB1 is known as an activator of EMT by transcriptionally suppressing E-cadherin expression, which not only promotes migration and invasion, but also is closely associated with the resistance to cancer chemotherapeutics in various cancer cells^41^.

In the present study, we found that the USP4/β-catenin axis was involved in metastatic potential through USP4-mediated stabilization of β-catenin and that knockdown of USP4 and β-catenin suppressed the metastatic potential including clonogenicity, migration, and invasion, and induced MET by downregulating ZEB1 expression. Luminescence imaging experiments indicated that knockdown of USP4 suppressed brain metastasis *in vivo* and promoted the overall survival and brain metastasis-free survival. In conclusion, we demonstrated that the USP/β-catenin axis confers metastatic ability to lung adenocarcinoma and is thus a promising target for brain metastatic lung cancer treatment.

## Methods

### Cell culture and transfection

Brain metastatic PC14PE6/LvBr4 cells were previously established through left ventricle (LV) injection of PC14PE6 lung adenocarcinoma cells^9^. For *in vivo* bio-imaging experiments, PC14PE6/LvBr5-Luc cells were generated by transducing pre-made Lentiviral Expression Particles for firefly luciferase (LVP325, Amsbio, Abingdon, UK) as previously described^9^. Parental PC14PE6 and its brain metastatic cells, PC14PE6/LvBr4 and LvBr5-Luc, were maintained at 37 °C and 5% CO_2_ in DMEM (Hyclone, Logan, UT, USA) supplemented with 10% fetal bovine serum (FBS) and 1% antibiotic-antimycotic solution (GIBCO-BRL, Grand Island, NY, USA).

Cells were plated at a density of 5 × 10^5^ cells/dish and transfected with control (CTRL) or indicated small interfering RNAs (siRNAs) using Lipofectamine2000 (Invitrogen, Carlsbad, CA, USA) according to the manufacturer’s instructions. Control and USP4-targeting siRNAs (USP4 siRNA #1: 5′-GGCUCUGGAACAAAUACAUUU-3′; #2: 5′-GGCUCUGGAACAAAUACAUUU-3′) were synthesized by ST Pharm (Seoul, South Korea). SiRNA for β-catenin was purchased from Santa Cruz Biotechnology (sc-29209; Santa Cruz, CA, USA). XAV939 was purchased from Sigma-Aldrich and solubilized with dimethyl sulfoxide (DMSO). PC14PE6/LvBr4 cells were treated with 10 μM XAV9393 (final DMSO concentration = 0.1%) for 24 h.

### Western blot and RT-qPCR analyses

For western blot analysis, whole cell lysates were prepared using RIPA buffer containing protease and phosphatase inhibitors (Roche, Basel, Switzerland). Equal amounts of lysate were separated by SDS-PAGE and transferred to polyvinylidene fluoride membranes (Millipore, Billerica, MA, USA). The membranes were blocked and incubated with primary antibodies overnight. After incubation with the appropriate secondary antibodies, membranes were visualized using enhanced chemiluminescence. Antibodies for β-catenin (610154; BD Biosciences, Franklin Lakes, NJ, USA), USP4 (A300-830A; Bethyl Laboratories, Montgomery, TX, USA), GAPDH (ab8245; Abcam, Cambridge, UK), E-cadherin (610181; BD Biosciences), and ZEB1 (A301-921A; Bethyl Laboratories) were used in this study.

To assess mRNA levels, real-time quantitative polymerase chain reaction (RT-qPCR) was performed using the following specific primers: β-catenin, forward: CTTGGACTGAGACTGCTGATCTTG, reverse: CACCAGAGTGAAAAGAACGATAGCTA; USP4, forward: CCTGGGCTCTGTGGACTTG, reverse: TGTTGATTTCGGCTTCATACTC; E-cadherin, forward: CGGGAATGCAGTTGAGGATC, reverse: AGGATGGTGTAAGCGATGGC; ZEB1, forward: AGCAGTGAAAGAGAAGGG, reverse: GGTCCTCTTCAGGTGCCT; SOX9, forward: CCCTTCGTGGAGGAGGCGGA, reverse: GGCCTGCAGCGCCTTGAAGA; JUN, forward: CAGGTGGCACAGCTTAAACA, reverse: GTTTGCAACTGCTGCGTTAG; VEGFA, forward: ATGACGAGGGCCTGGAGTGTG, reverse: CCTATGTGCTGGCCTTGGTGAG; EDN1, reverse: CAAGGAGCTCCAGAAACAGC, reverse: GTGGACTGGGAGTGGGTTTC; GAPDH, forward: TGCACCACCAACTGCTTAGC, reverse: GGCATGGACTGTGGTCATGAG.

### Determination of metastatic potential: migration and invasion

Metastatic potential was determined by assessing migratory and invasive activities. To assess migratory activity, a wound closure assay and Transwell cell migration assay (ECM508; QCM™ 24-well Colorimetric Cell Migration Assay, Millipore) were performed. For the *in vitro* wound closure assay, cells were cultured in 12-well plates until a monolayer had formed, and then a scratch was formed using pipette tips. After additional incubation for 24 h, the migrating distance was evaluated under a microscope. For the Transwell migration assay, cells were loaded into upper chamber in FBS-free media and migration was initiated by adding complete FBS-containing media to the lower chamber. Migrated cells were stained using crystal violet and migratory activity was assessed by measuring absorbance. Invasive activity was determined using BD BioCoat™ Matrigel Invasion Chamber (354480; BD Bioscience)^9^. After 24 h incubation, invaded cells were fixed with 100% methanol and stained with 0.1% hematoxylin and eosin. Invasive activity was determined by counting the number of invaded cells from more than 10 fields.

### Determination of β-catenin protein stability

To determine β-catenin protein stability, PC14PE6 and PC14PE6/LvBr4 cells were treated with 20 μg/ml cycloheximide (Sigma, St. Louis, MO, USA) and harvested at the indicated times. β-Catenin protein levels were determined by western blotting and quantified by image analysis. To assess the effect of USP4 silencing on β-catenin protein stability, transfected cells were treated with 40 μg/ml cycloheximide to stop protein synthesis.

### Determination of clonogenicity

For the clonogenicity assay, transfected cells (approximately 300 cells) were plated into a 6-well plate and cultured in complete medium for 2 weeks. After the cells were stained with 0.2% crystal violet, the number of stained colonies was counted from more than 3 independent wells.

### *In vivo* metastasis assay using bioluminescence imaging

All animal procedures were performed according to the Guide for the Care and Use of Laboratory Animals published by the National Institutes of Health (NIH) and the Animal Experiment Guidelines of Samsung Biomedical Research Institute (SBRI). All animal experiments were approved by the Institutional Animal Care and Use Committee (IACUC) of the Samsung Medical Center (SMC). To examine experimental metastasis *in vivo*, transfected PC14PE6/LvBr5-Luc cells (2 × 10^5^ in 100 μl of HBSS) were directly injected through the left ventricle (LV) of the heart. The body weights of the animals were measured daily, and an autopsy was performed immediately after 25% weight loss. Bioluminescence images were acquired using the IVIS Spectrum imaging system (PerkinElmer, Waltham, MA, USA) and the *in vivo* bioluminescence signal was quantified by measuring the photon flux (photons/s/cm^2^/steradian) within a region of interest drawn around the bioluminescence signal at the indicated times. Kaplan-Meier curves and *p*-values for overall survival and brain metastasis-free survival were analyzed in systemic metastasis models.

## Additional Information

**How to cite this article**: Hwang, S. J. *et al.* Ubiquitin-specific protease 4 controls metastatic potential through β-catenin stabilization in brain metastatic lung adenocarcinoma. *Sci. Rep.* **6**, 21596; doi: 10.1038/srep21596 (2016).

## Supplementary Material

Supplementary Information
