# Supplementary material for: Ubiquitin-specific protease 4 controls metastatic potential through β-catenin stabilization in brain metastatic lung adenocarcinoma
Source: Sci Rep. 2016 Feb 17;6:21596. doi: 10.1038/srep21596 (PMC4756316; doi:10.1038/srep21596)
Supplement: Supplementary Information [file srep21596-s1.pdf]

Supplementary information for SREP-15-28889 , Hwang et al.

Ubiquitin-specific protease 4 controls metastatic potential through β-catenin stabilization in brain metastatic lung adenocarcinoma

SJ Hwang, HW Lee, HR Kim, H Lee, CH Shin, SI Yun, DH Lee, DH Kim, KK Kim, KM Joo, HH Kim

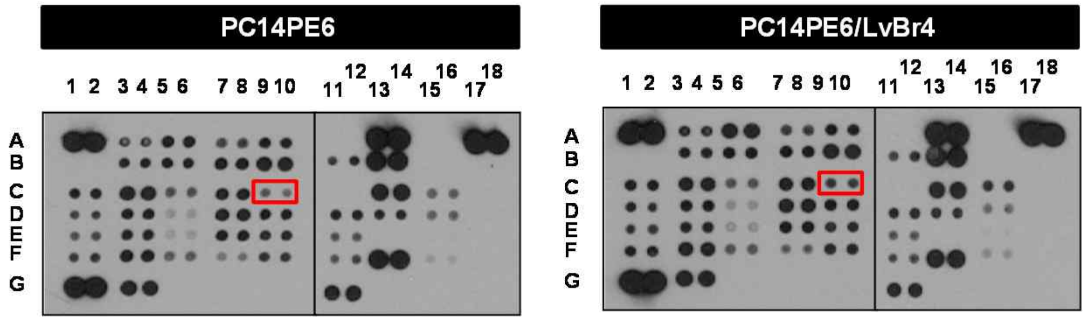

Human Phospho-Kinase Assay (Protepme Profiler^{TM}, R&D Systems)									
	1 2	3 4	5 6	7 8	9 10	11 12	13 14	15 16	17 18
A	Ref Spot	p38α	ERk1/2	JNK	GSK3α/β		p53		Ref Spot
B		EGFR	MSK1/2	AMPKα1	Akt	Akt	p53		
C	TOR	CREB	HSP27	AMPKα2	β-Catenin	p70S6K	p53	c-Jun	
D	Src	Lyn	Lck	STAT2	STAT5a	p70S6K	RSK1/2/3	eNOS	
E	Fyn	Yes	Fgr	STAT6	STAT5b	STAT3	p27	PLC-γ1	
F	Hck	Chk2	FAK	PDGF Rβ	STAT5a/b	STAT3	WNK1	PYK2	
G	Ref Spot	PRAS40				HSP60			PBS

Supplementary figure S1. Proteome analysis for searching differentially expressed signaling molecules. In order to investigate the molecular mechanism underlying higher metastatic potential of PC14PE6/LvBr4 cells, we have compared the level of various signaling molecules using phospho-kinases proteome profiler (Proteome Profiler^{TM} Human Phospho-Kinase Array Kit, R&D Systems, ARY003B). Briefly, cells were lysed with RIPA buffer and equal amounts of protein were incubated with according to the manufacturer's instruction.

Supplementary figure S2, Hwang et al.

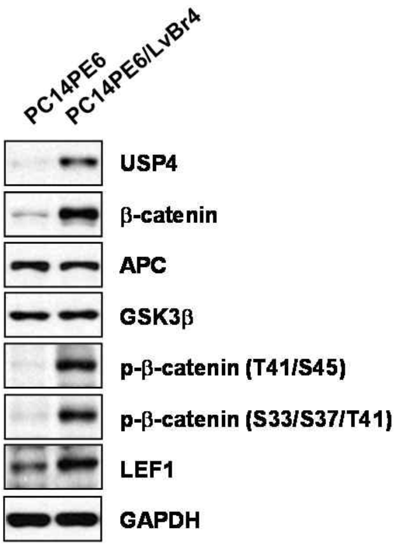

Supplementary figure S2. Destruction complex is not involved in increased expression of β-catenin in PC14PE6/LvBr4 cells. To examine whether increased expression of β-catenin is due to alteration of its destruction complex, the expression level of APC, GSK3β, and phosphorylated β-catenin was compared between parental PC14PE6 and brain metastatic PC14PE6/LvBr4 cells by Western blot.

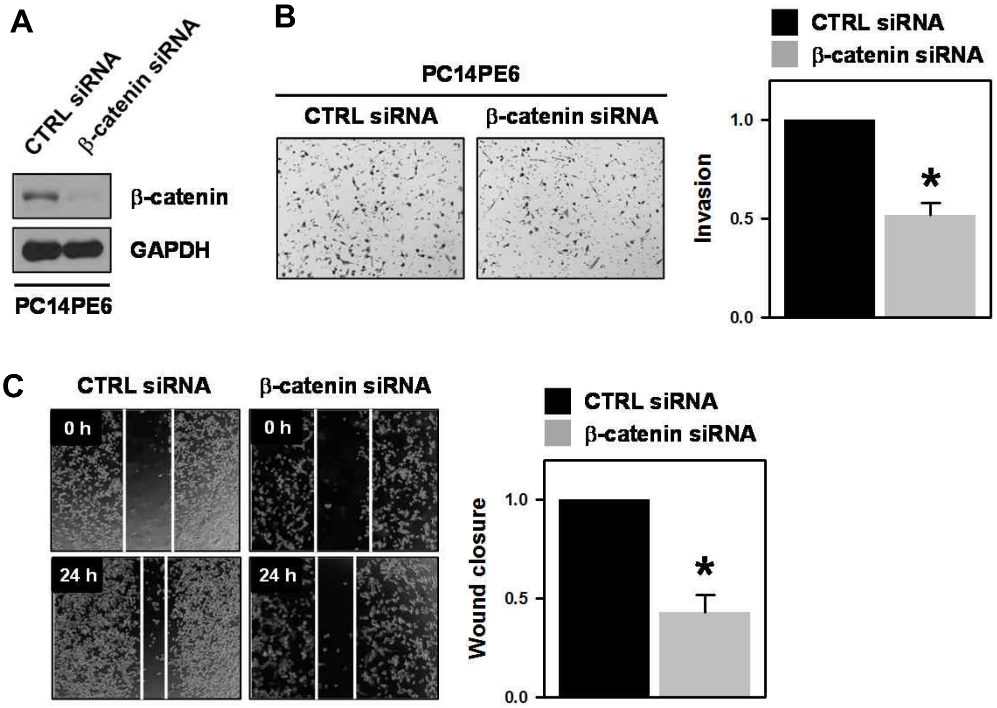

Supplementary figure S3. Knockdown of β-catenin inhibits the invasive and migratory abilities of PC14PE6 cells. To investigate the effect of b-catenin silencing on metastatic potential, PC14PE6 cells were transfected with control (CTRL) or β-catenin-specific siRNA. (A) The level of b-catenin was determined by Western blot. (B) Invasiveness was assessed using BD BioCoat™ Matrigel Invasion Chamber. After the invaded cells were fixed and stained as described in Materials and Methods, the invasive activity was determined by counting the number of invaded cells from more than ten fields. (C) Migratory ability was determined by wound closure assay. Transfected cells were cultured in 12-well plates until a monolayer had formed, and then a scratch was formed using pipette tips. After additional incubation for 24 h, the migrating distance was evaluated under a microscope. Data are means and standard deviation from more than three independent experiments. *, $p < 0.05$.

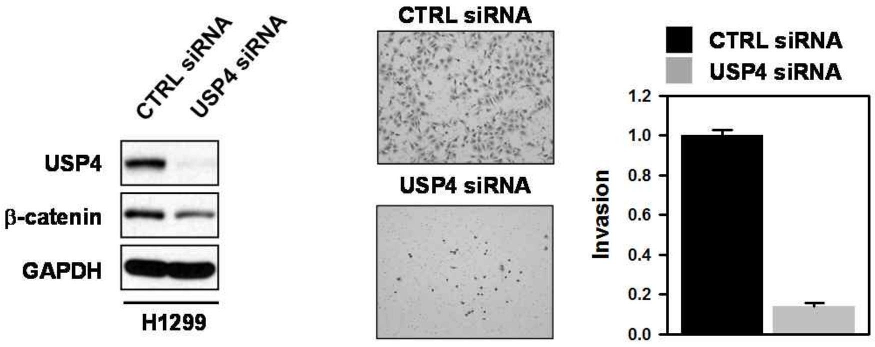

Supplementary figure S4. Knockdown of USP4 decreased the expression level of β-catenin and inhibited the invasive activity in H1299 lung cancer cells. To verify the effect of USP4 on β-catenin expression and invasiveness, H1299 cells were transfected with control (CTRL) or USP4-specific siRNA for 48 h. The level of USP4 and β-catenin was determined by Western blot (left panel). The effect of USP4 silencing on invasiveness was assessed using BD BioCoat™ Matrigel Invasion Chamber. After the invaded cells were fixed and stained as described in Materials and Methods, the invasive activity was determined by counting the number of invaded cells from more than ten fields.

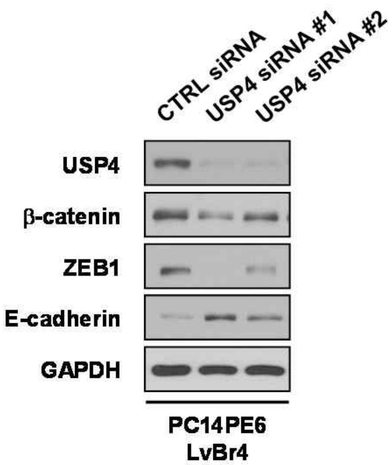

Supplementary figure S5. Knockdown of USP4 decreased the expression of β-catenin and inhibited EMT process via β-catenin/ZEB1 in PC14PE6/LvBr4 cells. To verify the effect of USP on the expression of β-catenin and EMT process, PC14PE6/LvBr4 cells were transfected with control (CTRL) or 2 independent USP4-specific siRNA for 48 h. The level of USP4 , β-catenin, ZEB1, and E-cadherin was determined by Western blot.

Supplementary figure S6, Hwang et al.

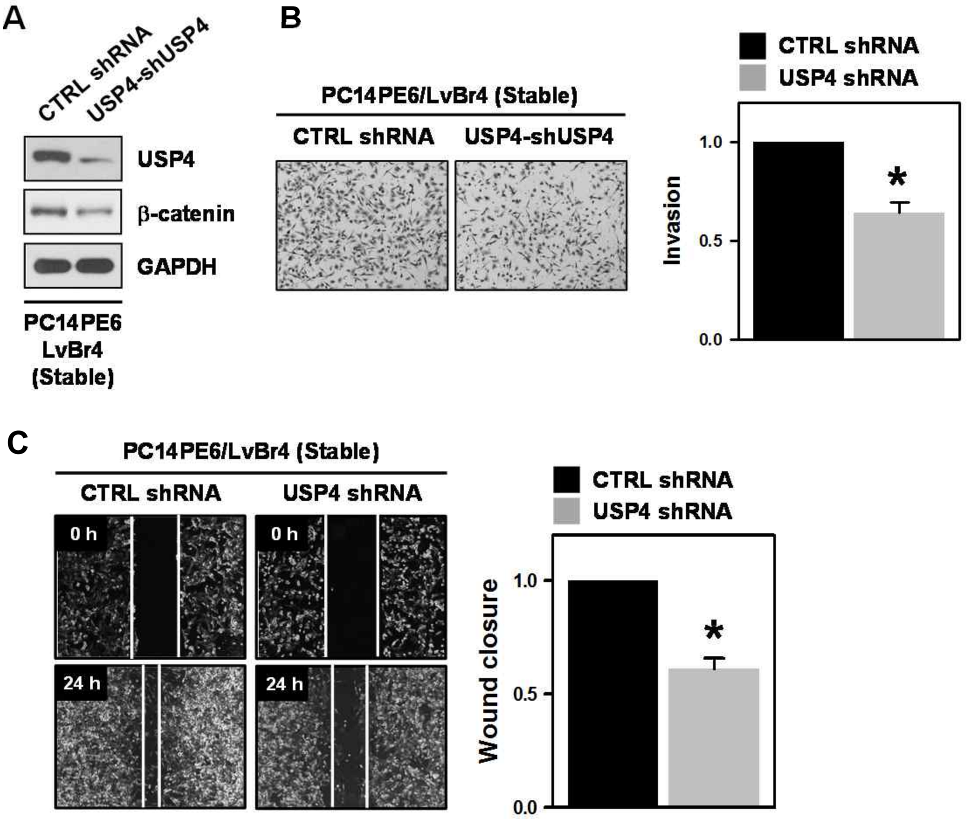

Supplementary figure S6. Stable knockdown of USP4 downregulates β-catenin and inhibits invasive and migratory activities in PC14PE6/LvBr4 cells. To verify the effect of USP on the β-catenin expression and invasiveness, PC14PE6/LvBr4 cells were transfected with control (CTRL) or USP4-specific shRNA and selected by treatment with puromycin for more than 2 weeks. (A) The level of USP4 and β-catenin was determined by Western blot. (B) Invasiveness was assessed using BD BioCoat™ Matrigel Invasion Chamber. After the invaded cells were fixed and stained as described in Materials and Methods, the invasive activity was determined by counting the number of invaded cells from more than ten fields. (C) Migratory ability was determined by wound closure assay. Stable cells were cultured in 12-well plates until a monolayer had formed, and then a scratch was formed using pipette tips. After additional incubation for 24 h, the migrating distance was evaluated under a microscope. Data are means and standard deviation from more than three independent experiments. *, $p < 0.05$.
